# Investigation of Individual Variability and Temporal Fluctuations in Exhaled Nitric Oxide (FeNO) Levels in Healthy Individuals

**DOI:** 10.3390/arm93040026

**Published:** 2025-07-21

**Authors:** Emi Yuda, Tomoki Ando, Yukihiro Ishida, Hiroyuki Sakano, Yutaka Yoshida

**Affiliations:** 1 Innovation Center for Semiconductor and Digital Future, Mie University, Tsu 514-0000, Japan; ando.tomoki@mytherapist.jp (T.A.); ishida@secondheart.co.jp (Y.I.); hirosakano@gmail.com (H.S.); yoshida.icsdf@mie-u.ac.jp (Y.Y.); 2 Department of Management Science and Technology, Graduate School of Engineering, Tohoku University, Sendai 982-0002, Japan

**Keywords:** exhaled nitric oxide (FeNO), airway inflammation, circadian rhythm, individual variability, repeated measures ANOVA

## Abstract

**Highlights:**

**What are the main findings?**

- Exhaled nitric oxide (FeNO) levels in healthy individuals show substantial inter-individual variability.
- Diurnal variation was observed, with greater fluctuations in the morning.
- No significant differences were found between time periods (*p* = 0.29).

**What is the implication of the main findings?**

- Timing and individual differences should be considered when using FeNO as a physiological marker in healthy subjects.

**Abstract:**

Measurement of nitric oxide (NO) concentration in exhaled breath (FeNO) is a quantitative, non-invasive, simple, and safe method for assessing airway inflammation. It serves as a complementary tool to other methods for evaluating airway diseases. However, little is known about the typical NO levels in healthy individuals, including individual differences and the influence of measurement timing. Therefore, this study classified measurement times into four periods and statistically analyzed NO levels in healthy individuals. The mean values among groups were compared using repeated measures ANOVA on six participants. The analysis showed large individual variations in NO levels, resulting in no significant difference (*p* = 0.29). Notably, greater fluctuations were observed in the morning. These findings align with previous studies suggesting the influence of circadian rhythms and the redundancy of repeated measurements. This study highlights the need to consider timing and individual variability when using FeNO as a physiological marker in healthy populations.

## 1. Introduction

Exhaled nitric oxide (FeNO) measurement is a quantitative, non-invasive, simple, and safe method for assessing airway inflammation. It serves as a valuable complementary tool alongside other diagnostic methods for evaluating airway diseases such as asthma [1,2,3,4,5,6]. Due to its simplicity and safety, FeNO testing has gained increasing attention in both clinical and research settings. However, despite its clinical potential, FeNO testing is not commonly performed in routine health assessments, resulting in limited knowledge about its behavior in healthy individuals. Most prior studies have focused on patients with respiratory diseases, particularly asthma, to explore the diagnostic and prognostic value of FeNO [7,8,9,10,11,12,13,14,15,16,17,18,19,20,21,22] (Table 1). As a result, normative data regarding FeNO levels in healthy populations remain scarce.

In the context of airway disease evaluation, multiple biomarkers and physiological indicators—including spirometry parameters, eosinophil counts, exhaled breath condensates, and imaging findings—have been proposed as prognostic factors. Nevertheless, FeNO stands out due to its unique capacity to directly reflect eosinophilic airway inflammation and to respond sensitively to anti-inflammatory treatment. This makes it particularly suitable for early detection, disease monitoring, and treatment responsiveness assessment, especially in asthma and related conditions. Therefore, incorporating FeNO into a broader panel of assessment tools can enhance diagnostic precision and prognostic accuracy.

Understanding the range of FeNO values in individuals without airway disease is essential for establishing reference standards. In particular, it is important to investigate both individual variability and temporal fluctuations throughout the day, which may be influenced by factors such as circadian rhythm, recent physical activity, and environmental exposure [23,24]. These variations must be taken into account to correctly interpret FeNO values and avoid misclassification in both research and clinical applications.

The aim of this study is to investigate FeNO levels in healthy individuals by analyzing individual differences and temporal changes. By classifying FeNO measurements into four distinct time periods and applying statistical analysis, we seek to characterize natural intra-individual and inter-individual variability in FeNO levels under normal physiological conditions. These findings are expected to contribute to the establishment of baseline FeNO profiles for healthy subjects and inform future use of FeNO as a reliable biomarker for airway status monitoring.

## 2. Materials and Methods

### 2.1. Participants

A total of 6 healthy participants (mean age: 61.3 ± 14 years, 2 females) were enrolled in this study. The inclusion criteria required all participants to be free from respiratory diseases. The exclusion criteria included individuals diagnosed with respiratory disorders (Table 1). In addition to medical history screening, a lifestyle questionnaire (https://www.pref.chiba.lg.jp/kenzu/press/2025/enq-r6.html accessed on 15 July 2025) was used to confirm the absence of atopic dermatitis, allergic rhinitis, and smoking habits, which are known to affect FeNO levels. The times at which NO was measured are shown in Table 2. All participants provided informed consent before participation. The study was approved by the Ethics Committee of the Faculty of Engineering, Mie University (Approval number 132, approved 19 February 2025). Although the sample size was limited to six individuals, this study was designed as an exploratory investigation to examine intra-individual and inter-individual variability in FeNO levels under controlled, healthy conditions. The relatively small sample size allowed for detailed, repeated measurements across multiple time points, facilitating the identification of temporal patterns and individual differences without confounding effects from underlying disease. This approach was suitable for the primary objective of establishing baseline FeNO characteristics in healthy subjects and was undertaken to aid in the design of future larger studies.

The measurement times were grouped as shown in the table below (Table 3).

### 2.2. FeNO Measurement Device

Exhaled nitric oxide (FeNO) was measured using a NIOX VERO device (Chest M.I., Inc., Tokyo, Japan; Medical Device Approval Number: 22700BZX00030000, JAN Code: 7350047030229). The device was operated via a PC application, “NIOX Panel,” with USB cable communication.

FeNO measurements followed the guidelines established by the American Thoracic Society (ATS) and the European Respiratory Society (ERS), which recommend exhaling at a constant flow rate of 50 mL/s ± 10% for 10 s. The measurement process involved the following steps: Participants inhaled NO-free gas through a built-in NO scrubber in the device’s respiratory handle. They then exhaled at a constant flow rate (50 mL/s ± 10%) for 10 s following ATS/ERS standards. The last 3 s of exhalation were sampled for FeNO analysis to ensure a stable reading. If the exhalation flow rate or duration was insufficient, the measurement was interrupted and repeated. The total measurement time, from initiation to result display, was approximately 1 min and 30 s. Regarding the output of NO concentration values, the measuring device samples the last 3 s of exhalation and analyzes the NO concentration. FeNO values are typically 15 ppb or less in healthy individuals, but values of 22 ppb or higher indicate a possibility of asthma, and values of 37 ppb or higher indicate a high likelihood of asthma [1,2,6].

Next, we discuss device calibration and environmental conditions. The NIOX VERO device measures FeNO in parts per billion (ppb) with high precision. To maintain measurement accuracy, the device, sensor, and respiratory handle have predefined expiration and usage limits. During this study, these components were replaced as necessary, eliminating the need for additional calibration. All measurements were conducted in a temperature-controlled environment (24 ± 2 °C). Participants remained seated at rest during the FeNO measurement process.

### 2.3. Exchange Kinetics of Exhaled Nitric Oxide (NO)

For an explanation of the exchange kinetics of NO in exhaled air, see Nikolaos M. et al. In this study [25], a model of the lung divided into two compartments, airways and alveoli, was constructed to reproduce the relationship between exhaled NO concentration and expiratory flow rate and to provide an index for assessing the contribution of NO sources in the lung. In this study, a two-compartment mathematical model was developed to analyze the exchange dynamics of NO in exhaled breath, dividing the lungs into two regions: the airways and the alveoli. Each compartment is surrounded by a tissue layer capable of producing and consuming NO, with adjacent blood flow (bronchial circulation for the airways and pulmonary circulation for the alveoli) assumed to be an infinite sink for NO. The NO production rate was estimated based on existing experimental data, and parameters were adjusted to match experimental results by simulating the relationship between the NO elimination rate at end-exhalation (ENO) and the exhalation flow rate (V˙E). Additionally, the relationship between ENO and V˙E was used as an index to evaluate the relative contributions of the airways and alveoli to exhaled NO. This model demonstrated that exhaled NO concentration is inversely correlated with exhalation flow rate and that both the airways and alveoli contribute to NO elimination. Unlike previous single-compartment models, which struggled to explain certain experimental findings, this two-compartment model successfully reproduces observed phenomena, providing a more comprehensive framework for understanding NO exchange dynamics [26,27,28,29,30,31]. The concentration of exhaled NO is known to depend on the V˙E, a phenomenon that must be accurately accounted for in mathematical models of NO exchange. As exhalation flow rate increases, the concentration of NO in exhaled breath generally decreases due to the shortened residence time of NO within the airways and the dilution effect of increased airflow. Conversely, at lower flow rates NO accumulates in the airway compartment, leading to higher concentrations in the exhaled breath. To quantify this relationship, the two-compartment model incorporates flow-dependent NO transport dynamics. The parameters governing NO production and uptake in the airway and alveolar compartments were estimated using experimental data, particularly the observed relationship between the NO elimination rate at ENO and V˙E. By fitting the model to experimental results, key physiological parameters such as NO production rates in the airways and alveoli, tissue diffusion coefficients, and airway wall uptake rates were optimized. Through this approach the model reproduces the characteristic inverse correlation between exhaled NO concentration and exhalation flow rate [32].

The NO concentration in exhaled breath is described by the following formula:

(1) $$C\;no\left(t\right)=C\;alv+\frac{J\;aw}{D\;aw+V\;exp}$$

where *Cno(t)* is the concentration of NO in exhaled air, *Calv* is the alveolar NO concentration, *Jaw* is the airway NO flux, and *Daw* is the airway diffusion parameter.

### 2.4. Statistical Analysis

We compared FeNO levels at different measurement points using repeated measures analysis of variance (ANOVA). We used SPSS Statistics (IBM SPSS Statistics v29.0.1) for the analysis. We evaluated the temporal variation in FeNO levels, taking into account the within-subject variability. The threshold for statistical significance was set at *p* < 0.05.

Mathematically, the repeated measures ANOVA model can be expressed as follows:

(2) $$Y_{ij}=\mu +\alpha_{j}+s_{i}+\varepsilon_{ij}$$

where

Y_ij_ is the FeNO value of subject i at time point j,

*μ* is the overall mean,

*α*_*j*_ is the fixed effect of the time point j,

*s*_*i*_ is the random effect associated with subject *i* (accounting for within-subject variability),

*ε*_*ij*_ is the residual error term.

The main hypothesis tested is as follows:

(3) $$H_{0}:\;\alpha_{1}=\alpha_{2}=\ldots =\alpha_{k}$$

against the following alternative:

(4) $$H_{1}:\;{At\;least\;one\;\alpha }_{j}\;differs\;(presence\;of\;time\;effect)$$

This model allows us to assess whether FeNO levels significantly vary across the measurement times within the same individuals (2–4).

## 3. Results

The results of statistical analysis using analysis of variance are shown in the table (Table 4, Table 5, Table 6 and Table 7). NO levels fluctuated greatly from person to person, so no significant differences were observed (Table 5). NO levels showed particularly large fluctuations in the morning. Statistical analysis was performed on the four groups of 11am−1pm, 1–3pm, and 3–5pm, and there were slight differences in the measured values depending on the measurement time (*p* = 0.088).

Data interval: This column indicates the time periods, typically in hours, during which data was measured. The intervals are presented in a “start time–end time” format (e.g., “8–9” for 8:00 to 9:00). Frequency: This column shows the count of measured data points that fall within each specified “Data Interval.” For example, between 9:00 and 10:00, 31 data points were recorded. Classification: This column appears to categorize or aggregate the “Frequency” data at certain intervals. For instance, the “80” value for the 10–11 interval likely represents a cumulative or grouped count related to the preceding “Frequency” values, or a separate classification count for that period. Similarly, “89,” “103,” and “33” are presented at specific intervals, while other rows are left blank, suggesting these are specific classification points. The “Total” row summarizes the data.

This table presents statistical analysis of exhaled nitric oxide (NO) concentrations, categorized into specific two-hour time classifications. Number of measurements: This row indicates the total count of individual exhaled NO measurements taken within each respective time classification. For example, there were 80 measurements taken between 9:00 and 11:00. Time classification: This row defines the specific two-hour intervals during which the exhaled NO measurements were analyzed. The classifications are 9–11 (9:00–11:00), 11–13 (11:00–13:00), 13–15 (13:00–15:00), and 15–17 (15:00–17:00). Mean (ppb): This row shows the average (mean) concentration of exhaled NO in parts per billion (ppb) for each time classification. For instance, the average exhaled NO between 13:00 and 15:00 was 33.02 ppb. S.D. (standard deviation): This row provides the standard deviation for the exhaled NO concentrations within each time classification. Standard deviation measures the amount of variation or dispersion of a set of values. A higher S.D. indicates a wider spread of NO values around the mean. S.E. (standard error): This row presents the standard error of the mean for each time classification. Standard error estimates the variability between sample means, indicating how accurately the sample mean represents the true population mean. A smaller S.E. suggests a more precise estimate of the population mean.

This table offers a detailed statistical insight into the exhaled NO profiles of individual participants. Participant ID: This column identifies each unique individual included in the analysis, ranging from 1 to 6. Mean (ppb): This column displays the average (mean) concentration of exhaled NO, measured in parts per billion (ppb), for each respective participant. For instance, Participant 1 had an average exhaled NO concentration of 30.45 ppb. S.D. (standard deviation): This column shows the standard deviation of exhaled NO measurements for each participant. Standard deviation indicates the spread or variability of an individual’s NO readings around their mean. A higher S.D. suggests greater fluctuation in NO levels for that participant.

CV (%) (coefficient of variation): This column presents the coefficient of variation, expressed as a percentage, for each participant’s exhaled NO measurements. The CV is calculated as (Standard Deviation/Mean) × 100. It is a normalized measure of dispersion, allowing for comparison of variability between different participants, even if their mean values differ significantly. A higher CV indicates greater relative variability in NO measurements for that participant.

This table presents descriptive statistics for nitric oxide (NO) measurements across different categories, followed by the results of a one-way analysis of variance (ANOVA). Category: Represents the different groups or classifications of the NO measurement data. Frequency: The number of data points (measurements) within each respective category. Mean: The average NO concentration (likely in ppb, as per previous tables) for each category. S.D. (standard deviation): A measure of the dispersion or spread of NO values around the mean within each category. S.E. (standard error): An estimate of the variability of the sample mean, indicating the precision of the mean for each category. 95% CI (95% confidence interval): A range of values within which the true population mean is estimated to lie with 95% confidence. Min: The minimum NO concentration observed within each category. Max: The maximum NO concentration observed within each category. Total: Provides the overall summary statistics for all combined measurements across all categories. The lower part of the table presents the results of a one-way ANOVA, which is used to determine if there are any statistically significant differences between the means of three or more independent (unrelated) groups. In this context, it assesses whether the mean NO concentrations across the different “Categories” (groups) are significantly different from each other. Sum of squares: Measures the total variation in the data. Between groups: Represents the variation between the means of the different categories. Within groups: Represents the variation within each category (also known as error sum of squares). df (degrees of freedom): Indicates the number of independent values that can vary in a data set.

## 4. Discussion

In this study, we analyzed exhaled NO values using analysis of variance for 80 cases from 9 to 11 a.m., 89 cases from 11 to 1 p.m., 103 cases from 1 to 3 p.m., and 33 cases from 3 to 5 p.m. The results showed that there were no significant differences because NO fluctuates greatly from person to person, and that there are large fluctuations in the morning. First, we will consider the effects of circadian rhythms. In a previous study, the possible effects of the important internal variable circadian rhythm on exhaled breath temperature (EBT) were analyzed in a group of 24 healthy adult volunteers [33,34]. EBT was measured repeatedly at different times of day (8 a.m., noon, 4 p.m., and 8 p.m.), and the correlation with axillary temperature readings at these times was analyzed. The results reported that there were significant differences in some axillary temperature readings. The highest EBT was reported at 4 p.m., and the lowest EBT was reported at 8am, indicating that circadian rhythms affect EBT. This is the first analysis of circadian rhythms in healthy subjects exhaled NO levels, and it was shown that they are not affected by circadian rhythms compared to EBT.

Next, we will consider repeated measurements. Recent studies have shown excellent reproducibility in FeNO measurements, and in a study that determined whether repeated FeNO measurements were necessary in the same session for asthma screening the value of repeated measurements was shown to be insignificant [35]. The results of this study are also consistent with the results of previous studies, as although there is a large individual difference in the measured NO values of healthy individuals the variation within individuals is not that large.

This study has several limitations. First, the sample size is relatively small, which may affect the generalizability of the results. A limited number of participants can reduce the statistical power required to detect significant differences, potentially impacting the interpretation of the findings. As a result of calculating the correlation matrix from the NO measurement data at four time points for each subject and calculating the approximate statistical power, in this case, with six subjects, four time points, and a significance level of 0.05, the power to detect differences in observed NO levels due to time was moderate (65%) when the correlation between repeated measures was assumed to be 0.5. Additionally, there was a wide age range among the participants, which is another limitation. Variations in age can influence the production and fluctuations of FeNO, and age-related physiological differences in the respiratory system may introduce bias in the results. The impact of these age-related variations should be considered, and a more age-homogeneous sample would have been preferable for more precise analysis. This study was limited to healthy subjects and did not include subjects with respiratory diseases, so it is not possible to clearly show the extent of clinically significant changes. And, in this study, we did not measure NO concentrations at flow rates other than 50 mL/s, so we could not obtain information on Jaw and Daw.

Given these constraints, future studies should involve larger sample sizes and aim to balance age groups to obtain more reliable and accurate results. However, despite these limitations, this study holds significant value for several reasons. Firstly, it represents an attempt to analyze diurnal variation using data from healthy individuals, which is a relatively novel approach in the field. By examining how FeNO fluctuates across different times of the day we have provided insights into the natural variability of FeNO levels. Moreover, the study highlights the individual differences in FeNO measurements, an important factor that has often been overlooked in previous research. Understanding these individual variations can be crucial in interpreting FeNO data, particularly when considering its potential for use in clinical applications such as asthma diagnosis or remote monitoring [36,37,38,39].

## 5. Conclusions

In this study we analyzed the NO levels (total 305 cases) of six subjects measured at four different times in order to clarify the existence of diurnal variations in NO levels in healthy individuals and to examine the applicability of NO measurements to telemedicine. The results of the analysis showed that NO levels varied greatly between individuals, with no significant differences between groups. In addition, it was revealed that NO levels showed particularly large variations in the morning. These results differ from previous studies showing diurnal variations in breath temperature. Further research is needed to clarify the factors behind individual differences.

## Figures and Tables

**Table 1:** Previous studies on NO measurement.

Author(s)	Year	Journal	FeNO Measurement Method	Subjects
Stewart & Katial [7]	2012	*Immunol. Allergy Clin. North Am.*	General review of FeNO	Asthma and allergic disease patients
Hoyte, Gross & Katial [8]	2018	*Immunol. Allergy Clin. North Am.*	General update/review of FeNO	Asthma patients
García-Río et al. [9]	2011	*J. Asthma.*	Two-compartment model	Asthma patients
Wyszyńska et al. [10]	2022	*Molecules*	Review of measurement devices (e.g., NIOX)	Not specified
Pontier et al. [11]	2014	*Nitric Oxide*	Offline chemiluminescence	Divers (healthy)
Baraldi & Carraro [12]	2006	*Paediatr. Respir. Rev.*	FeNO and breath condensate	Children with respiratory conditions
Popov et al. [13]	2017	*J. Breath Res.*	FeNO with exhaled breath temperature	Respiratory disease patients
Amann et al. [14]	2014	*Annu. Rev. Anal. Chem.*	General review including FeNO	Various diseases
Tonacci et al. [15]	2019	*Int. J. Psychol.*	Breath analysis (FeNO)	Individuals under stress
Minh, Blake & Galassetti [16]	2012	*Diabetes Res. Clin. Pract.*	Breath biomarkers including FeNO	Diabetes patients
Paleczek & Rydosz [17]	2022	*J. Breath Res.*	Algorithm review for FeNO in diabetes detection	Diabetes patients
Emilsson et al. [18]	2023	*Respir. Res.*	Exhaled biomarkers including FeNO	Adults with chronic cough
Peng et al. [19]	2016	*Sci. Rep.*	Fast non-equilibrium dilution IMS	Not clearly specified
Zitt [20]	2017	*J. Allergy Clin. Immunol. Pract.*	Fractional FeNO (fENO)	Asthma patients
MacBean et al. [21]	2018	*Physiol. Meas.*	Offline FeNO with dilution	Not specified
Zhang et al. [22]	2017	*Medicine (Baltimore)*	Meta-analysis of FeNO	Sleep apnea patients

**Table 2:** Medical exclusion criteria.

	Conditions
1	Subject with a history of respiratory disease (asthma, COPD, interstitial lung disease, etc.).
2	Subject with acute respiratory tract infections (cold, influenza, pneumonia, etc.).
3	Subject with lung cancer or severe lung disease.
4	Subject with recent airway inflammation.
5	Subject using bronchodilators (β2 agonists) or steroids.
6	Smokers or subject exposed to passive smoking.

**Table 3:** Measured time period and number of measurements.

Measurement Time	Number of Data
9–11	80
11–13	89
13–15	103
15–17	33

Participants in the experiment took the breath gas test to measure NO at a time convenient for them, so the amount of data varies depending on the time period.

**Table 4:** Number of measured data.

Data Interval	Frequency	Classification
8–9	5	
9–10	31	
10–11	49	80
11–12	69	
12–13	20	89
13–14	56	
14–15	47	103
15–16	27	
16–17	6	33
17–18	2	
18–19	13	
19–20	0	
20–21	3	
21–22	1	
22–23	1	
Total	330	305

**Table 5:** Analysis results of exhaled NO by time period.

Number of Measurements	80	89	103	33
Time classification	9–11	11–13	13–15	15–17
Mean (ppb)	31.43	29.82	33.02	28.09
S.D.	19.60	13.21	13.77	8.80
S.E.	2.19	1.40	1.36	1.53

**Table 6:** Participant NO measurement statistics.

Participant ID	Mean (ppb)	S.D.	CV (%)
1	30.45	2.78	9.14
2	29.78	15.29	51.36
3	25.29	10.64	42.08
4	27	14.79	54.77
5	33.46	14.45	43.19
6	30.12	12.93	42.92

**Table 7:** NO measurement summary and ANOVA.

Category	Frequency	Mean	S.D.	S.E.	95% CI	Min	Max
1	80	31.43	19.61	2.19	27.06–35.79	1	107
2	89	29.82	13.21	1.4	27.04–32.60	8	93
3	103	33.02	13.77	1.36	30.33–35.71	6	123
4	33	28.09	8.8	1.53	24.97–31.21	1	39
Total	305	31.13	15	0.86	29.44–32.82	1	123
	**Sum of Squares**		**Df**	**Mean Square**		**F**	**Sig.**
Between groups	832.126		3	277.375		1.236	0.297
Within groups	67,569.362		301	224.483			
Total	68,401.489		304				

## Data Availability

The data supporting the reported results in this study are available upon request by contacting the corresponding author via email. Access to the data is restricted to research purposes only.
